# Effect of a p38 MAPK inhibitor on FFA-induced hepatic insulin resistance *in vivo*

**DOI:** 10.1038/nutd.2016.11

**Published:** 2016-05-02

**Authors:** S Pereira, W Q Yu, J Moore, Y Mori, E Tsiani, A Giacca

**Affiliations:** 1 Department of Physiology, University of Toronto, Toronto, ON, Canada; 2 Department of Health Sciences, Brock University, St Catherines, ON, Canada; 3 Department of Medicine, Showa University School of Medicine, Shinagawa, Tokyo, Japan; 4 Department of Medicine, University of Toronto, Toronto, ON, Canada; 5 Institute of Medical Science, University of Toronto, Toronto, ON, Canada; 6 Banting and Best Diabetes Centre, University of Toronto, Toronto, ON, Canada

## Abstract

The mechanisms whereby prolonged plasma free fatty acids elevation, as found in obesity, causes hepatic insulin resistance are not fully clarified. We herein investigated whether inhibition of p38 mitogen-activated protein kinase (MAPK) prevented hepatic insulin resistance following prolonged lipid infusion. Chronically cannulated rats were subdivided into one of four intravenous (i.v.) treatments that lasted 48 h: Saline (5.5 μl min^−1^), Intralipid plus heparin (IH, 20% Intralipid+20 U ml^−1^ heparin; 5.5 μl min^−1^), IH+p38 MAPK inhibitor (SB239063) and SB239063 alone. During the last 2 h of treatment, a hyperinsulinemic (5 mU kg^−1^ min^−1^) euglycemic clamp together with [3-^3^H] glucose methodology was carried out to distinguish hepatic from peripheral insulin sensitivity. We found that SB239063 prevented IH-induced hepatic insulin resistance, but not peripheral insulin resistance. SB239063 also prevented IH-induced phosphorylation of activating transcription factor 2 (ATF2), a marker of p38 MAPK activity, in the liver. Moreover, in another lipid infusion model in mice, SB239063 prevented hepatic but not peripheral insulin resistance caused by 48 h combined ethyloleate plus ethylpalmitate infusion. Our results suggest that inhibition of p38 MAPK may be a useful strategy in alleviating hepatic insulin resistance in obesity-associated disorders.

## Introduction

Elevated plasma free fatty acids (FFAs), as found in obesity, induce hepatic insulin resistance.^1, 2, 3^ Prolonged exposure of hepatocytes to FFAs activates p38 mitogen-activated protein kinase (MAPK), which decreases the ability of insulin to reduce gluconeogenesis.^4^ In murine models of obesity, hepatic p38 MAPK is activated and when p38 MAPK is overexpressed in the liver, impairment of insulin signalling ensues.^5^ Nevertheless, the role of p38 MAPK in FFA-induced hepatic insulin resistance *in vivo* has not been assessed. We herein used prolonged (48 h) lipid infusion, namely Intralipid plus heparin (IH) infusion in rats^3^ and combined ethyloleate plus ethylpalmitate infusion in mice, to elevate plasma FFAs because thus far, p38 MAPK activation has been associated with prolonged *in vitro* exposure to FFAs or obesity-associated insulin resistance, a chronic model of FFA elevation. We have found that protein kinase C (PKC)-δ is activated in the liver after prolonged lipid infusion,^3^ and studies in hepatocytes have shown that PKC-δ activates p38 MAPK.^6^ Hence, in the current study we used a p38 MAPK inhibitor to determine whether it prevented hepatic insulin resistance caused by prolonged plasma FFA elevation.

## Materials and methods

### Experiments

The Animal Care Committee of the University of Toronto approved all procedures, which were in accordance with the Canadian Council of Animal Care Standards. Chronically cannulated^3^ female Wistar rats were randomized in a non-blinded manner into one of four intravenous (i.v.) treatments: Saline (SAL, 5.5 μl min^−1^), IH (20% Intralipid+20 U ml^−1^ heparin; 5.5 μl min^−1^), IH+p38 MAPK inhibitor SB239063 (SB (Sigma, St Louis, MO, USA); 2.25 mg kg^−1^ h^−1^ for first hour and 0.55 mg kg^−1^ h^−1^ thereafter^7^) and SB alone. After an overnight fast, at 44 h of treatment, [3-^3^H] glucose was started (8 μCi bolus plus 0.15 μCi min^−1^). A 2-h hyperinsulinemic (insulin infusion: 5 mU kg^−1^ min^−1^) euglycemic clamp^3^ was initiated at 46 h. Blood samples for plasma assays were collected during the basal period (30 min before the clamp) and during the last 30 min of the clamp. For western blot analysis, the liver was collected under anesthesia after 48 h of infusions.

Male C57BL6 mice underwent a hyperinsulinemic (5 mU kg^−1^ min^−1^) euglycemic clamp with [3-^3^H] glucose at the end of 48 h infusion of ethanol control in glycerol vehicle (EtOH, 0.12 μmol min^−1^) or combined ethyloleate+ethylpalmitate infusion in a 2:1 ratio in glycerol vehicle (EtO/P, total dose: 0.12 μmol min^−1^) or EtO/P+SB (4.5 mg kg^−1^ during the first hour, 1.1 mg kg^−1^ h^−1^ thereafter). This lipid infusion method is based on the conversion of ethyl fatty acids to fatty acids and ethanol by plasma esterases.^8^

### Assays and calculations

Measurements of plasma glucose, insulin and FFA and calculations of glucose kinetics were performed as reported previously.^3^ For western blots, cytosolic fractions or whole homogenates of liver samples were prepared.^3, 9^ The primary antibody for total activating transcription factor 2 (ATF2) was from Santa Cruz Biotechnology (Santa Cruz, CA, USA; cat #sc-187), while the rest of primary antibodies used were from Cell Signaling Technology (Danvers, MA, USA; cat #9221, 9251, 9252, 9271, 9272).

### Statistics

Data are means±s.e.m. Significance was accepted when *P*<0.05. One-way ANOVA followed by Tukey's *t*-test was used, unless further specified. Sample size was based on variances obtained in our previous studies using this model and is described in figure legends. Variances were similar across groups.

## Results

Plasma glucose was not different among groups during the basal (SAL: 6.60±0.18 mm, *n*=7; IH: 6.06±0.22, *n*=8; IH+SB: 5.77±0.39, *n*=6; SB: 6.66±0.06, *n*=5) and clamp periods (SAL: 6.40±0.20; IH: 6.06±0.28; IH+SB: 5.83±0.39; SB: 6.49±0.06). Basal plasma FFAs were ~2-fold higher (*P*<0.05) in IH-infused groups (SAL: 472±31 μm; IH: 894±62; IH+SB: 1105±103; SB: 557±85). During the hyperinsulinemic clamp, FFAs were lower than basal as expected but remained elevated (*P*<0.05) in IH-infused groups (SAL: 110±23 μm; IH: 549±70; IH+SB: 514±67; SB: 176±38). There was no intergroup difference in basal (SAL: 67±12 pm; IH: 102±20; IH+SB: 118±32; SB: 119±39) or clamp plasma insulin (SAL: 506±29 pm; IH: 522±25; IH+SB: 569±58; SB: 557±59).

**Figure 1:**
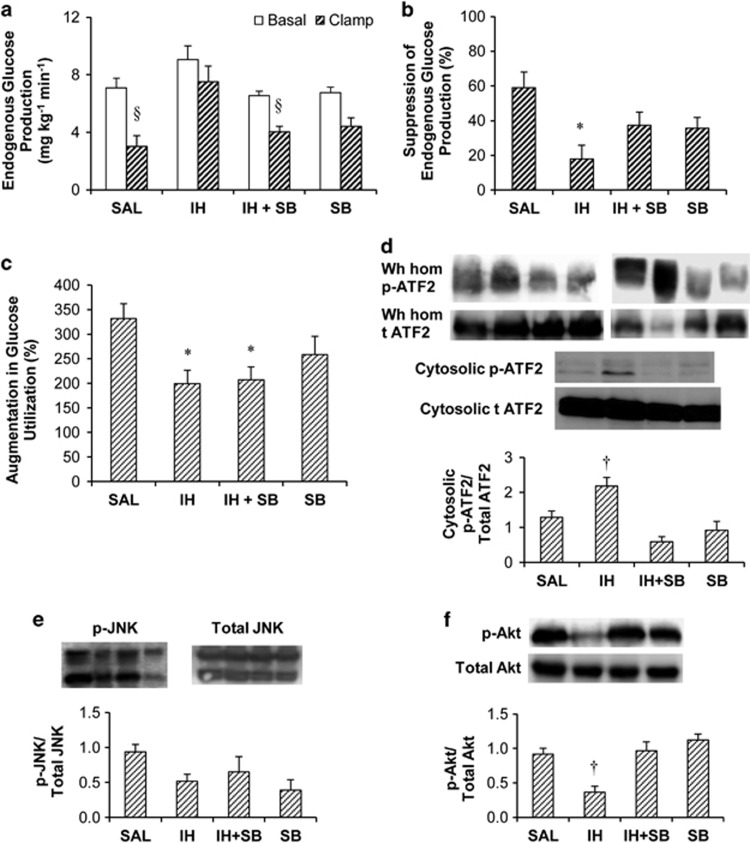
(**a**) Endogenous glucose production during basal and clamp periods. (**b**) Percent suppression of endogenous glucose production by insulin. (**c**) Percent augmentation of glucose utilization by insulin. (**d**) Protein content of phosphorylated ATF2 (p-ATF2) relative to total ATF2 in liver cytosolic fractions and representative blots of protein content of p-ATF2 relative to total (t) ATF2 in cytosolic fractions and whole liver homogenates (Wh hom; performed to confirm the initial results in cytosolic fractions). (**e**) Protein content of phosphorylated c-jun N-terminal kinase (p-JNK) relative to total JNK in whole liver homogenates. (**f**) Protein content of p-Akt relative to total Akt in whole liver homogenates. Liver tissue collected after 48 h of treatment infusion without the hyperinsulinemic euglycemic clamp was used for (**d,** **e**). For (**f**), liver tissue collected after 48 h of treatment infusion and hyperinsulinemic euglycemic clamp was used. Data are means±s.e.m. Treatments: SAL, Saline; IH, Intralipid plus heparin; IH+SB, IH plus SB239063 (p38 MAPK inhibitor); SB, SB239063. For (**a**–**c**), *n*=7 for SAL, *n*=8 for IH, *n*=6 for IH+SB and *n*=5 for SB. For (**d**), *n*=5 for SAL, *n*=4 for IH, *n*=5 for IH+SB and *n*=3 for SB. For (**e**), *n*=4 for SAL, IH and IH+SB, and *n*=3 for SB. For (**f**), *n*=4 for SAL, *n*=5 for IH, *n*=4 for IH+SB and *n*=4 for SB. *P<0.05 vs SAL. ^†^*P*<0.05 vs other groups. ^§^*P*<0.05 vs IH.

There was a trend for IH to increase basal endogenous glucose production, which did not reach significance (Figure 1a) and was abolished by SB. IH elevated endogenous glucose production during the clamp and SB prevented this elevation (Figure 1a). Insulin-induced suppression of endogenous glucose production (that is, hepatic insulin sensitivity) was blunted by IH and rescued by SB (Figure 1b). Glucose infusion rate and glucose utilization were higher (*P*<0.05) in SAL (27.0±1.3 mg kg^−1^min^−1^ and 30.0±1.2 mg kg^−1^min^−1^) than in IH (17.6±0.9 and 25.1±1.0), IH+SB (16.1±1.1 and 20.2±1.3) and SB alone (19.7±2.3 and 24.2±1.8). Percent augmentation in glucose utilization (that is, peripheral insulin sensitivity) was decreased by IH, which was not prevented by SB (Figure 1c).

As a marker of p38 MAPK activity, we determined the phosphorylation of its direct target ATF2 in liver tissues collected after 48 h of infusions, without hyperinsulinemic clamps because members of the MAPK family can be activated by insulin. IH increased ATF2 phosphorylation and this was prevented by SB (Figure 1d). In contrast, phosphorylation of c-jun N-terminal kinase (JNK), another MAPK implicated in insulin resistance, did not differ across groups (Figure 1e). In liver samples collected after clamp, phosphorylation (that is, activation) of Akt, a mediator of insulin signalling, was decreased by IH and this was prevented by SB (Figure 1f).

**Figure 2:**
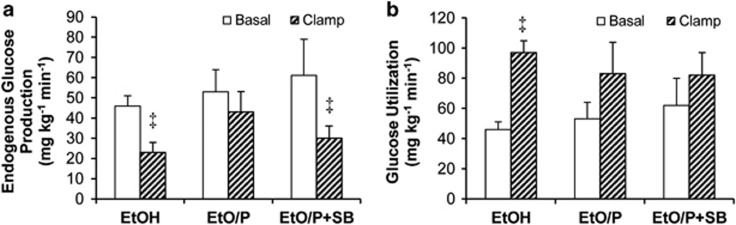
Glucose turnover results of studies in mice. Mice were infused for 48 h with: ethanol control in glycerol vehicle (EtOH), combined ethyloleate+ethylpalmitate infusion in a 2:1 ratio in glycerol vehicle (EtO/P) or EtO/P+SB239063 (SB). (**a**) Endogenous glucose production during basal and clamp periods is shown. (**b**) Glucose utilization during basal and clamp periods is shown. Data are means±s.e.m. Repeated measures two-way ANOVA followed by Tukey's *t*-test were used; ^‡^*P*<0.05 for clamp vs basal for a given treatment. *n*=6 for EtOH, *n*=5 for EtO/P and *n*=3 for EtO/P+SB.

To corroborate our data, we used another model of prolonged (48 h) lipid infusion, combined ethyloleate plus ethylpalmitate infusion with or without SB in mice, and found that again, SB prevented lipid-induced hepatic but not peripheral insulin resistance (Figure 2).

## Discussion

We found herein that the p38 MAPK inhibitor SB239063 prevented hepatic insulin resistance caused by prolonged plasma FFA elevation in rats. This effect was associated with decreased activation of ATF2, a direct target of p38 MAPK, in the liver. We confirmed the benefits of SB239063 on lipid-induced hepatic insulin resistance in mice infused with combined ethyloleate plus ethylpalmitate. SB239063, however, did not have beneficial effects on peripheral insulin sensitivity.

To our knowledge, this is the first study to show that inhibition of p38 MAPK prevents hepatic insulin resistance caused by FFAs *in vivo*. The role of p38 MAPK in FFA-induced hepatic insulin resistance has been studied in hepatocytes,^4^ where p38 MAPK stabilized PTEN (protein phosphatase and tensin homolog deleted on chromosome 10), an antagonist of the effects of phosphoinositide 3-kinase.^4^ Akt is downstream of phosphoinositide 3-kinase and the current study shows that the p38 MAPK inhibitor prevents IH-induced impairment in hepatic Akt activation. FFA-induced p38 MAPK activation also increases gluconeogenic gene transcription,^6^ which may explain the trend toward increased basal endogenous glucose production by IH, abolished by SB239063.

IH increased ATF2 phosphorylation and SB239063 thwarted this, which suggests that SB239063 acts by inhibiting p38 MAPK. SB239063 inhibits both p38α MAPK and p38β MAPK^7^ although p38α MAPK mediated the FFA effects in hepatocytes.^4, 6^ In contrast, activation of hepatic JNK was not altered by IH or SB239063.

SB239063 did not improve peripheral insulin sensitivity. p38 MAPK is activated in muscle of humans with type 2 diabetes,^10, 11^ and of mice following lipid infusion;^12^ however, p38 MAPK inhibition did not improve insulin-stimulated muscle glucose uptake.^11^ There are reports that SB239063 inhibits glucose uptake in muscle cell lines^13^ perhaps independent of p38 MAPK,^14^ and our results suggest that SB239063 alone decreased glucose utilization *in vivo*. However, the percent augmentation in glucose utilization by insulin infusion did not differ in SB239063 vs SAL.

In contrast to our results, one study has found that hepatic p38 MAPK activation, via adenoviral-mediated overexpression of MAPK kinase 6, in *ob/ob* mice is beneficial to insulin sensitivity.^15^ Our results support the results by another group that using adenoviral-mediated overexpression of dominant-negative p38α MAPK found improved glucose tolerance and reduced hyperinsulinemia and PEPCK expression in *ob/ob* mice.^5^

In conclusion, in our models of prolonged lipid infusion, inhibition of p38 MAPK ameliorates hepatic insulin sensitivity. As prolonged lipid infusion activates liver PKC-δ^3^ and PKC-δ activates p38 MAPK in hepatocytes,^6^ PKC-δ may be upstream of p38 MAPK in FFA-induced hepatic insulin resistance *in vivo*, but this remains to be determined. The reason why p38 MAPK inhibition is effective on FFA-induced hepatic but not peripheral insulin resistance is unclear; however, there are different possibilities, including different mechanisms of insulin resistance in the periphery vs liver after 48 h IH^16^ and a possible adverse effect of SB239063 on glucose utilization.

As some MAPK inhibitors can be used in humans and are in phase II clinical trials for their antinflammatory and antineoplastic properties,^17, 18, 19^ it would be of interest to determine whether these inhibitors can improve obesity-associated hepatic insulin resistance.
